# Complete mitochondrial genome of *Hemiptelea davidii* (Ulmaceae) and phylogenetic analysis

**DOI:** 10.1080/23802359.2019.1644562

**Published:** 2019-07-23

**Authors:** Hua-Bo Liu, Yang Zhao, Xing-Ze Zhang, Chao Sun, Ji-Chen Xu

**Affiliations:** a National Engineering Laboratory for Tree Breeding, Beijing Forestry University, Beijing, China;; b Forest Seedling Station of Yantai City, Yantai, China;; c State Key Laboratory of Tree Genetics and Breeding, Research Institute of Forestry, Chinese Academy of Forestry, Beijing, China

**Keywords:** *Hemiptelea davidii*, mitochondrial genome, phylogenetic analysis

## Abstract

*Hemiptelea davidii* (Hance) Planch is a potential valuable forest tree in arid sandy environments. Here, the complete mitochondrial genome of *H. davidii* was assembled using a combination of the PacBio Sequel data and the Illumina Hiseq data. The mitochondrial genome is 460,941 bp in length, including 37 protein-coding genes, 19 tRNA genes, and three rRNA genes. The GC content of the whole mitochondrial genome is 44.84%. Phylogenetic analyses indicated that *H. davidii* is close with *Cannabis* and *Morus* species.

*Hemiptelea davidii* (Hance) Planch belonging to *Hemiptelea* of Ulmaceae is a kind of shrub or small tree with a height up to 10 m, which mainly distributed in China. It could survive well in arid sandy environment and was then recognized as a potential valuable forest tree resource in low rainfall region (Bai et al. 2008). Despite the complete chloroplast genomes for *H. davidii* have been reported in our previous study, the genetic information available for this species is still extremely scarce (Liu et al. 2019). Plant mitochondria plays essential role to supply the cell with metabolic energy and was also involved in resisting adverse circumstances. Thus, we report the complete mitochondrial genome of *H. davidii* here in order to better understand their characteristics in response to the species status. The annotated mitochondrial genome has been deposited into GenBank under accession number MN061667.

In this study, the plant material were harvested from Yantai of Shandong Province (geographic coordinates: 37°9′50″N, 121°25′58″E), China and the specimen were conserved at Shandong Provincial Center of Forest Tree Germplasm Resources (voucher number: HD2016003). The mitochondria DNA was separated from fresh leaves using an improved method according to Chen et al. (2011) and then sequenced on the Illumina Hiseq 4000 platform and PacBio Sequel platform, respectively. The mitochondrial genome was assembled using a combination of the PacBio Sequel data and the Illumina Hiseq data using SPAdes v3.10.1 software (Antipov et al. 2016). The genome sequences were annotated using GeSeq (Tillich et al. 2017).

The *H. davidii* mitochondrial genome is assembled into a single circular-mapping molecule of 460,941 bp, with a GC content of 44.84%. The mitochondrial genome contains a total of 59 genes, including 37 protein-coding genes, 19 tRNA genes, and three rRNA genes. Of the protein-coding genes, *nad5* is the largest gene (2016 bp) while *atp9* is the smallest gene (225 bp).

Sequences of 10 common protein-coding genes (*cox1*, *cox2*, *cox3*, *matR*, *nad2*, *nad4*, *nad6*, *nad7*, *nad9*, and *rps3*) were aligned with the homologous genes in other 13 species using MAFFT (Katoh and Standley 2013). Phylogenetic analyses were performed using maximum-likelihood (ML) with RAxML based on GTRGAMMA model with 1000 bootstrap replicates (Silvestro and Michalak 2012). The phylogenetic tree showed that *H. davidii* is close with *Cannabis* and *Morus* species (Figure 1), which is consistent with the evolutionary relationship of species.

**Figure 1.**
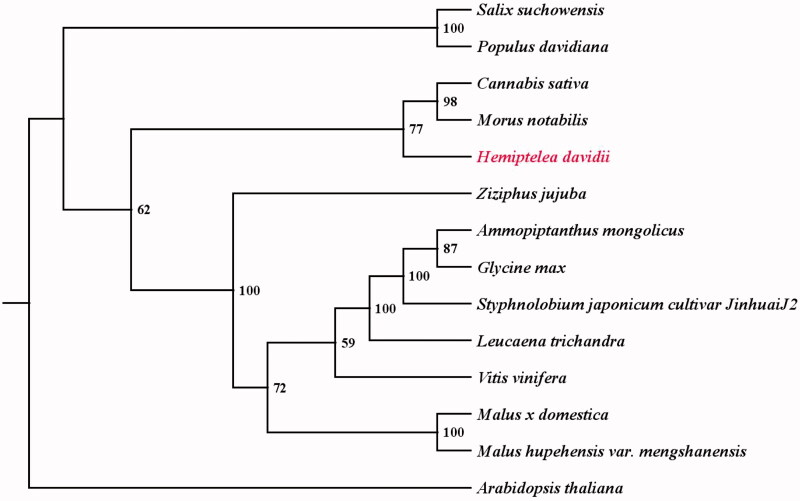
The phylogenetic tree of 14 plant mitochondrial genomes based on 10 common protein-coding genes using *Arabidopsis thaliana* as an out-group. Accession number: *Ammopiptanthus mongolicus* (MF683210), *Arabidopsis thaliana* (Y08501), *Cannabis sativa* (KU310670), *Glycine max* (JX463295), *Hemiptelea davidii* (MN061667), *Leucaena trichandra* (MH717173), *Malus hupehensis var. mengshanensis* (KR534606), *Malus x domestica* (NC_018554), *Morus notabilis* (MK301435), *Populus davidiana* (KY216145), *Salix suchowensis* (KU056812), *Styphnolobium japonicum cultivar JinhuaiJ2* (MG757109), *Vitis vinifera* (FM179380), and *Ziziphus jujuba* (KU187967). The number on each node indicates the bootstrap value.
